# Impact of ultraviolet radiation on filtering facepiece respirators and SARS-CoV-2 detection

**DOI:** 10.3389/fpubh.2025.1537742

**Published:** 2025-03-06

**Authors:** Khaled Al-Hadyan, Najla Al-Harbi, Sara Bin Judia, Maha Al-Ghamdi, Ibtihaj Alsharif, Layla Alharbi, Maha Al-Mozaini, Belal Moftah, Salem Al-Ghamdi, Ghazi Alsbeih

**Affiliations:** ^1^Department of Biomedical Physics, Research and Innovation, King Faisal Specialist Hospital and Research Centre (KFSH&RC), Riyadh, Saudi Arabia; ^2^Department of Infection and Immunity, Research and Innovation, KFSH&RC, Riyadh, Saudi Arabia; ^3^Medical Physics Unit, Gerald Bronfman Department of Oncology, McGill University, Montreal, QC, Canada; ^4^Infection Control and Hospital Epidemiology Department, KFSH&RC, Riyadh, Saudi Arabia; ^5^College of Medicine, Alfaisal University, Riyadh, Saudi Arabia

**Keywords:** COVID-19, SARS-CoV-2, filtering facepiece respirators, decontamination ultraviolet irradiation, RT-qPCR, filtration efficiency

## Abstract

**Introduction:**

The global coronavirus disease (COVID-19) pandemic of 2020 caused by SARS-CoV-2 resulted in a shortage of filtering facepiece respirators (FFRs), such as N95 and KN95 masks. Ultraviolet-C (UV-C) irradiation has been proposed as a potential decontamination method to enable FFR reuse and mitigate the shortage. This study aims to evaluate the impact of UV-C irradiation on the filtration efficiency (FE) of various FFR types and SARS-CoV-2 RNA degradation.

**Method:**

We evaluated the effect of UV-C irradiation (60–900 mJ/cm^2^) on the FE using various particle sizes (PSs, 0.3–5 μm) representing seven common FFR types (3M-8210, 3M-1860, Gerson 1730, Medline, Benehal, KN95 “duck shape,” and KN95 “molded shape”) and the stability of the SARS-CoV-2 RNA genes (*E, RdRp2, RdRp4*, and *N*) using reverse transcription quantitative real-time polymerase chain reaction (RT-qPCR).

**Results:**

Following UV-C exposure, the FE of the FFRs at all PSs was >98%, with no significant differences among them (*p* > 0.05). UV-C irradiation significantly increased the RT-qPCR cycle threshold values (ΔCt) for the *E*, *RdRp2*, and *RdRp4* SARS-CoV-2 genes (*p* ≤ 0.001) compared with the control, indicating marked RNA degradation; however, it did not significantly affect *N* gene stability (*p* = 0.612).

**Discussion:**

These results support the use of UV-C as an effective decontamination technique for FFRs, particularly during periods of shortage.

## 1 Introduction

Throughout history, infectious diseases with pandemic potential have emerged and spread regionally or globally. The recent pandemic has exposed a long-standing weakness in the medical supply chain, which has resulted in personal protective equipment (PPE) shortages, price hikes, and disruptions in trade routes (1). Governments and businesses scramble for solutions, often in vain. A shortage of sufficient PPE supplies is a major problem because they are required to prevent the spread of the disease (2).

The recent coronavirus disease (COVID-19) caused by severe acute respiratory syndrome coronavirus 2 (SARS-CoV-2) was declared by the World Health Organization (WHO) as a global pandemic on March 11, 2020 (3). As of March 31, 2024, >774 million confirmed cases and > 7 million deaths have been reported worldwide (4). Filtering facepiece respirators (FFRs), including N95 masks, play an important role in healthcare systems by blocking SARS-CoV-2 airborne transmission between patients with COVID-19 and healthcare practitioners (5).

Fomite transmission is one of several recognized routes of SARS-CoV-2 transmission, involving the transfer of large infectious respiratory particles (IRPs) emitted by infected individuals through coughing, sneezing, or speaking onto contaminated surfaces, which others then contact (6–9). Another significant transmission route is airborne transmission, where smaller IRPs remain suspended in the air for extended periods and are inhaled in poorly ventilated environments (9–11).

During the peak of the COVID-19 pandemic in 2020, the rapid increase in COVID-19 cases caused a dramatic global shortage of FFRs, particularly N95 masks (12, 13). Therefore, decontamination and subsequent reuse of these masks are recommended to address the shortage.

There are six well-characterized N95 decontamination procedures, including vapor hydrogen peroxide, ethylene oxide, moist heat incubation, microwave oven, ultraviolet-C (UV-C), and gamma irradiation (GIR) (14–20). These methods have logistical and technical challenges, including a small capacity, limited penetration, a high risk of pathogen cross-contamination, and alterations in the physical characteristics of the mask material, which lead to adverse effects on filtration efficiency (FE), airflow, or fit test of the mask (19–24).

In March 2020, the National Center for Immunization and Respiratory Diseases, Centers for Disease Control (CDC), issued guidelines for the decontamination and reuse of FFRs to assist healthcare institutions in managing the global shortage of FFRs, particularly N95 masks, during the COVID-19 pandemic (25). The CDC considers UV-C as a promising N95 decontamination procedure. This method can simultaneously decontaminate N95 masks from pathogens and maintain the FE and physical stability of the N95 masks (25). Consistent with the CDC recommendations, several studies have identified UV-C (ranging between 200 and 280 nm) as a promising decontamination method for N95 masks using various approaches to validate decontamination, FE assessment, sterilization techniques, and UV-C sources (26–31).

UV-C inactivates pathogens by damaging their nucleic acids, specifically DNA and RNA, by forming crosslink between adjacent nucleic acid residues (32–35). For RNA pathogens, UV-C absorbed by RNA can induce RNA photoproducts (e.g., cyclobutane pyrimidine dimers), which disrupts their ability to replicate (36, 37). In addition, UV-C light causes RNA strand breaks due to the high energy associated with the UV-C light (38, 39). The minimum UV-C dose (also known as fluence) required to inactivate pathogens depends on the irradiation wavelength, the radiation sensitivity of the pathogen, and the environment of the pathogen during irradiation (e.g., air, surfaces, or media) (35, 40).

In this study, we determined the effect of UV-C on the FE of seven common FFRs. We examined the ability of UV-C to sterilize infected FFRs by detecting SARS-CoV-2 RNA following UV-C exposure using a reverse transcription quantitative real-time polymerase chain reaction (RT-qPCR) assay.

## 2 Materials and methods

### 2.1 Filtering facepiece respirators

Table 1 presents the FFRs used in this study. Briefly, seven types of FFRs were evaluated: 3 M-8210, 3 M-1860, Gerson 1730, Medline (cone style), Benehal (particulate respirator face mask), KN95 (duck shape), and KN95 (molded shape). The first five masks listed (all of the N95 type) were approved by the USA National Institute for Occupational Safety and Health (NIOSH), whereas two of the KN95 masks (duck and molded shapes) comply with Chinese standards for FFRs (41). Although NIOSH has not approved KN95 masks as FFRs, they are recommended for use in medical settings when there are shortages of NIOSH-approved masks (42). The FE of all FFRs was assessed before and after UV-C exposure, as outlined in Section 2.2, using a portable particle counter integrated with a custom-designed air duct, described in detail in Section 2.3.1.

**Table 1:** Description of FFRs used in the study.

FFRs	Type	Company	Country	Lot number	NIOSH approved
3 M-8210	N95	3 M	Multinational corporation	A13311	Yes
3 M-1860	N95	3 M	Multinational corporation	B15792	Yes
Gerson 1730	N95	Louis M. Gerson	United States	TC-84A-0160	Yes
Medline	N95	Medline	United States	TC-84A-5411	Yes
Benehal	N95	Suzhou Sanical Protective Product	China	541,529	Yes
KN95 (duck shape)	KN95	Yuyao Yukang Medical Equipment	China	20,200,506	No^1^
KN95 (molded shape)	KN95	ZhongShan XiaoLan YiShuai Gament Factory	China	2,020,042,701	No^1^

1 Meets Chinese standards GB2626:2006.

### 2.2 Ultraviolet-C irradiation source

**Figure 1:**
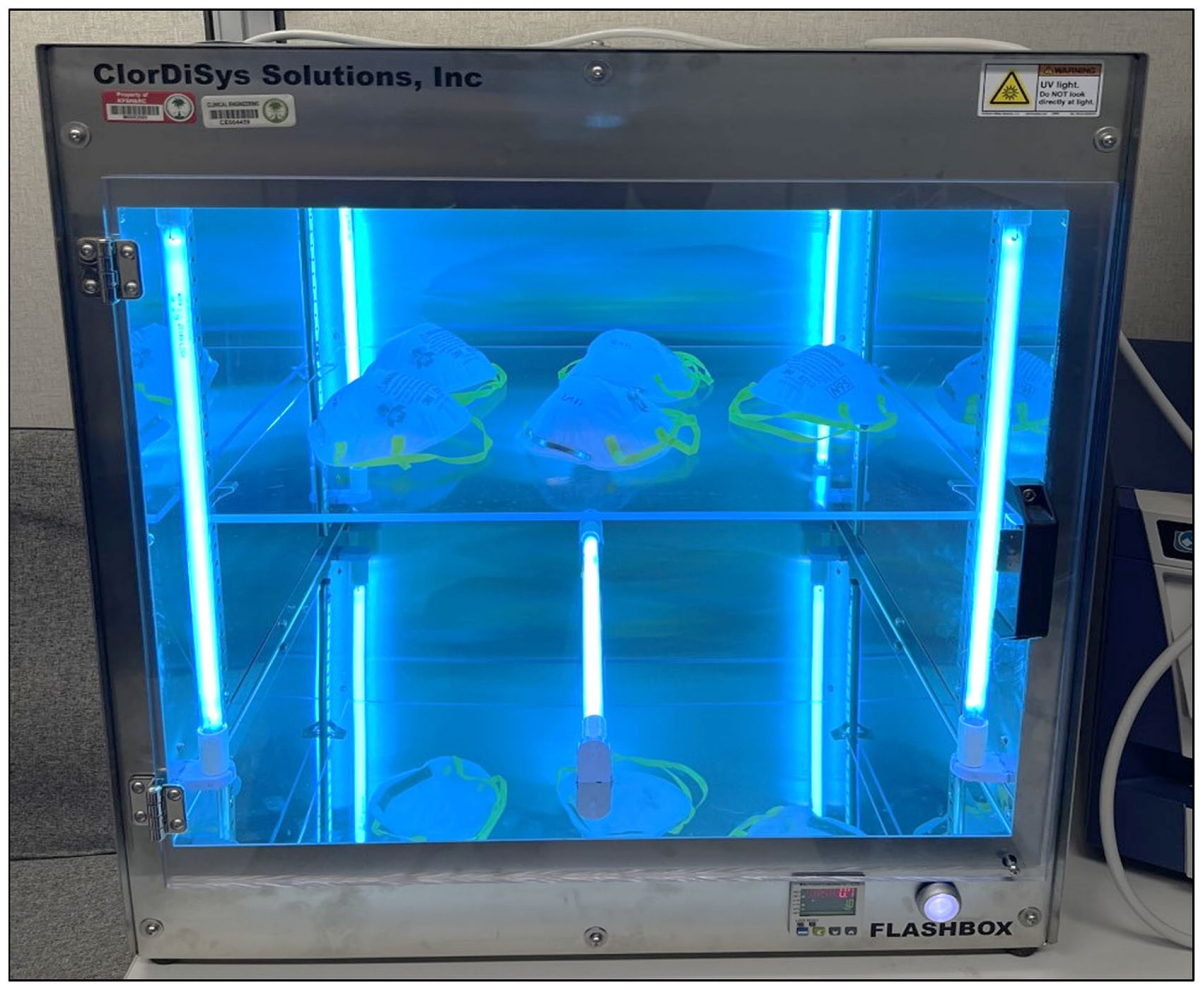
Flash Box UV-C disinfection chamber (ClorDiSys). A representative image showing five 3 M-8210 N95 masks undergoing UV-C irradiation in a Flash Box UV-C disinfection chamber.

A Flash Box UV-C disinfection chamber (ClorDiSys) was used in this study located at the Radiation Biology Section, Biomedical Physics Department, King Faisal Specialist Hospital and Research Centre (KFSH&RC), Riyadh, Saudi Arabia. The chamber was equipped with six UV-C bulbs, collectively providing an average UV-C output of 60 mJ/cm^2^ per minute. The internal dimensions of the chamber were 18.5 inches in width, 23 inches in depth, and 14 inches in height, which could easily fit five FFRs for each UV-C dose (Figure 1).

### 2.3 Filtration efficiency assessment of FFRs

#### 2.3.1 FE measurement

The FE measurement was performed according to an in-house method described previously (19, 20, 43). Briefly, a custom-designed air duct was manufactured to evaluate the FE of all seven FFR types using particulate matter (PM) in the air as particles. The air duct was connected to an AeroTrak particle counter (TSI, Model 9,306) that counts particle sizes (PSs) of 0.3, 0.5, 0.7, 1, 2, and 5 μm for 1-minute with a flow rate of 2.8 L/min.

#### 2.3.2 FE assessment of FFRs following ultraviolet-C irradiation

Five FFRs for each mask type underwent an FE assessment at each PS. Initially, the FE for each FFR was assessed without UV-C exposure (control, 0 mJ/cm^2^). Next, the FE of each FFR type was evaluated after each UV-C dose (60, 120, 180, 240, and 300 mJ/cm^2^). The particle number concentration of PM in the air was assessed at least five times before the FFR assessment. The average FE for each FFR was calculated after each UV-C dose for each PS using the following formula:


$$FE\;\left(\%\right)=100-\left(\frac{\mathrm{number}\;\mathrm{of}\;\mathrm{penetrated}\;\mathrm{particles}\;}{\mathrm{average}\;\mathrm{number}\;\mathrm{of}\;\mathrm{PM}\;\mathrm{in}\;\mathrm{the}\;\mathrm{air}}\times 100\right)$$

#### 2.3.3 Detection of SARS-CoV-2 RNA following ultraviolet-C irradiation

The stability of five SARS-CoV-2 RNA samples following exposure to various UV-C doses (0, 60, 300, 600, and 900 mJ/cm^2^) was evaluated using an RT-qPCR assay targeting the envelope (*E*), RNA-dependent RNA polymerase 2 (*RdRp2*), RNA-dependent RNA polymerase 4 (*RdRp4*), and nucleocapsid protein (*N*) SARS-CoV-2 genes.

#### 2.3.4 SARS-CoV-2 sample collection and ethical considerations

This study was approved by the Institutional Review Board at KFSH&RC, Riyadh, Saudi Arabia (RAC Approval# 2200047). The data from this study was approved for publication by the Research Affairs Department at KFSH&RC (clearance for publication# 2245329, dated 02/07/2024). Nasopharyngeal swabs were obtained from patients with COVID-19 between May 2020 and July 2020 for diagnosis and archived as part of another RAC-approved project (RAC Approval# 2200031) at KFSH&RC. Requirement for informed consent was waived due to the use of anonymized archived samples for research. Five SARS-CoV-2-positive samples were randomly retrieved, anonymized, coded, and used in this study. All methods were performed in accordance with the relevant guidelines and regulations.

#### 2.3.5 Sample processing and RNA extraction

Sample processing and RNA extraction was performed according to a previously described protocol (20). Briefly, five nasopharyngeal swabs were submerged in viral transport medium for diagnostic analysis. Aliquots of the leftover samples were stored at −80°C until viral RNA extraction in a biosafety level-3 research laboratory. Viral RNA extraction was performed using an in-house automated RNA extraction protocol (44).

#### 2.3.6 Effect of ultraviolet-C on SARS-CoV-2 RNA detection using an RT-qPCR assay

For each gene tested, five SARS-CoV-2 RNA samples were removed from a −80°C freezer, thawed, and aliquoted into six test tubes: two tubes were designated positive controls without UV-C (0 mJ/cm^2^) and stored at either room temperature (for 2 h) or at −80°C for the duration of the experiment, whereas four tubes were exposed to four different UV-C doses of 60, 300, 600, and 900 mJ/cm^2^, equivalent to exposure times of 1, 5, 10, and 15 min, respectively. The average UV-C output of the Flashbox UV-C disinfection chamber was 60 mJ/cm^2^ per minute. Two positive control tests performed at room temperature and at −80°C were used to assess the effect of a 2-h room temperature incubation on the stability of SARS-CoV-2 RNA using RT-qPCR. The lack of a cooling system in the Flash Box UV-C disinfection chamber required verification that the 2-h incubation at room temperature, necessary for completing the experiment, including sample preparation and UV-C irradiation, did not compromise RNA stability and detectability. In addition, a no-template control well, which contained all the reaction components except the RNA sample, was added to each RT-qPCR experiment as a negative control to ensure that any detected signal was not the result of non-specific amplification or contamination from reagents.

The TaqPath™ COVID-19 CE-IVD RT-PCR kit (Thermo Fisher Scientific: A48102) was used as previously described (45). The primer sets for the *E*, *RdRp2*, and *RdRp4* SARS-CoV-2 genes were selected to detect SARS-CoV-2 RNA based on WHO and CDC recommendations for human testing and diagnosis (Supplementary Table 1), and were adapted from the Charité Institute of Virology, Pasture Institute, Paris, France (46). The primers for the *N* gene were selected based on WHO recommendations adapted from the Department of Medical Sciences, Ministry of Public Health, Thailand (47). The conditions for performing RT-qPCR and the final concentrations of the reagents used are listed in Supplementary Tables 2, 3, respectively. The results are expressed as cycle threshold values (ΔCt), defined as the thermal cycle number at which the fluorescent signal exceeds that of the background and passes the threshold for positivity (48). The lower the ΔCt value, the higher the quantity of viral genetic material (viral load) in the sample.

### 2.4 Statistical analysis

A paired *t*-test was used to determine the overall statistical differences in the initial FE between the two 3 M N95 masks (3 M-8210 and 3 M-1860). A *t*-test was used to test for significant differences in the initial FE between various PSs within each mask and to examine the statistical differences in the mean ΔCt values between RNA samples following incubation for 2 h at room temperature and − 80°C. The parametric one-way repeated measures analysis of variance (RM-ANOVA) was used to assess significant differences in the mean FE between irradiated and non-irradiated masks, and in the mean ΔCt values between irradiated and non-irradiated CoV-SARS-2 RNA samples. Bonferroni’s t-test was used to correct for pairwise multiple comparisons in the RM-ANOVA, where appropriate. All statistical analyses were performed using SigmaPlot version 14.5 for Windows (SPSS Inc., Chicago, IL, USA). A *p*-value <0.05 was considered significant.

## 3 Results

### 3.1 FE of FFRs following ultraviolet-C irradiation

**Figure 2:**
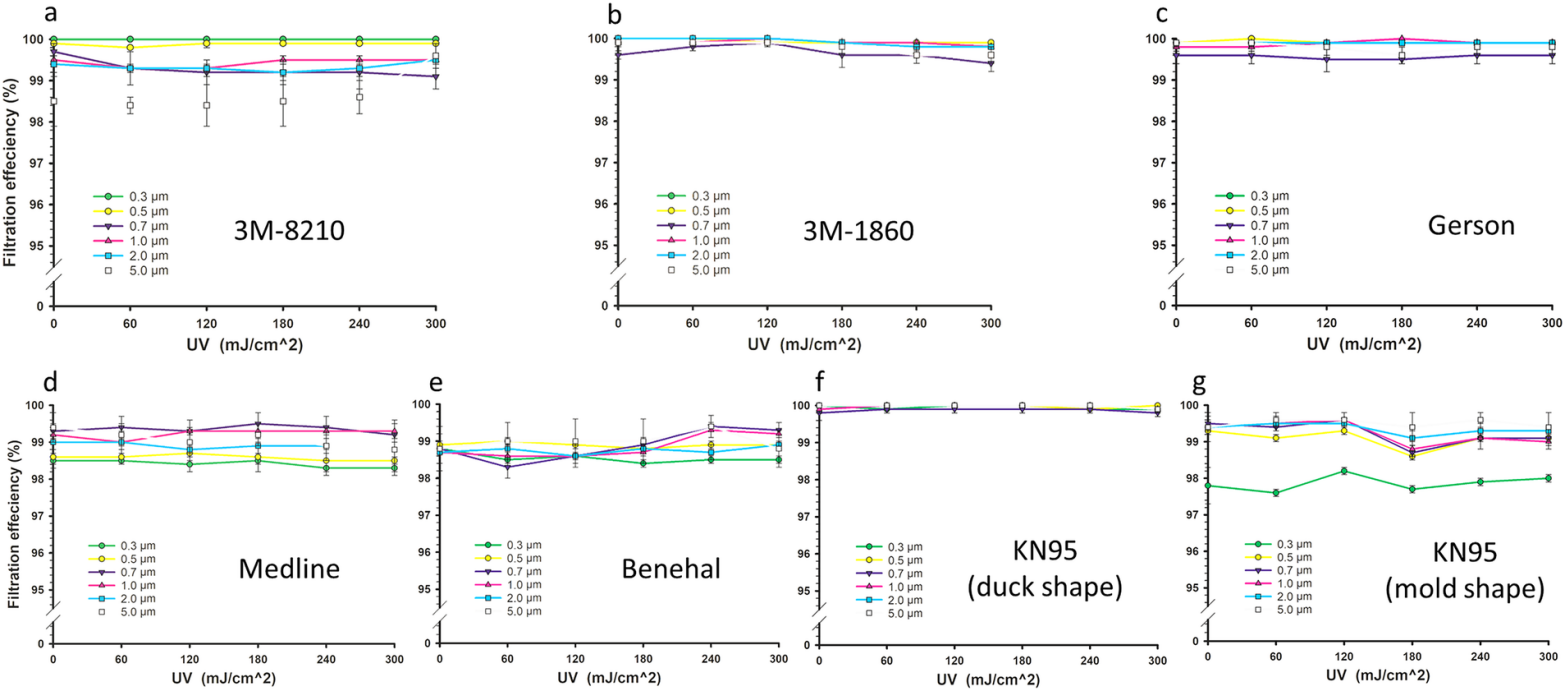
Filtration efficiency (FE) of filtering facepiece respirators (FFRs). FE of 3 M-8210 **(A)**, 3 M-1860 **(B)**, Gerson 1730 **(C)**, Medline **(D)**, Benehal **(E)**, KN95 “duck shape” **(F)**, and KN95 “molded shape” **(G)** for different particle sizes (0.3–5 μm) following various UV-C doses (0, 60, 120, 180, 240, and 300 mJ/cm^2^). Statistical analysis showed no significant difference (*p*-values ≥0.384) between irradiated FFRs and the control group as determined by a one-way repeated measures analysis of variance. Symbols represent the mean FE values of five FFRs, and error bars indicate the standard error.

The mean initial FE (0 mJ/cm^2^) measurements for all FFRs were > 98%, ranging between 98.8 and 99.9%, with no significant difference observed among them (*p* > 0.05) (Figure 2). The mean initial FE measurements for all PSs of 3 M-8210, 3 M-1860, Gerson, Medline, Benehal, KN95 (duck shape), and KN95 (molded shape) were 99.5%, with a standard error (SE) = 0.2, 99.9%; SE = 0.1, 98.8%; SE = 0.1, 99.0; SE = 0.2, 98.8%; SE = 0.3, 99.1%; SE = 0.2, and 99.9%; SE = 0.1, respectively.

The FE assessments of all FFR masks were assessed following UV-C doses of 0, 60, 120, 180, 240, and 300 mJ/cm^2^ at a PS of 0.3, 0.5, 0.7, 1, 2 and 5 μm (Figure 2). UV-C irradiation had no effect on the FE for all FFR types tested (Figure 2). The mean FEs at all PSs for the irradiated FFRs were not significantly different from the control group, with *p*-values ranging 0.384–1 (RM-ANOVA). The pairwise comparisons analysis between FE values for different PSs and UV-C doses were not considered as initial one-way repeated measures; ANOVA revealed no significant differences. Although no changes were observed in the structure of the FFR types following UV-C exposure, a burned odor was evident following UV-C irradiation, particularly at higher doses.

### 3.2 SARS-CoV-2 RNA stability following ultraviolet-C irradiation using RT-qPCR assay

**Figure 3:**
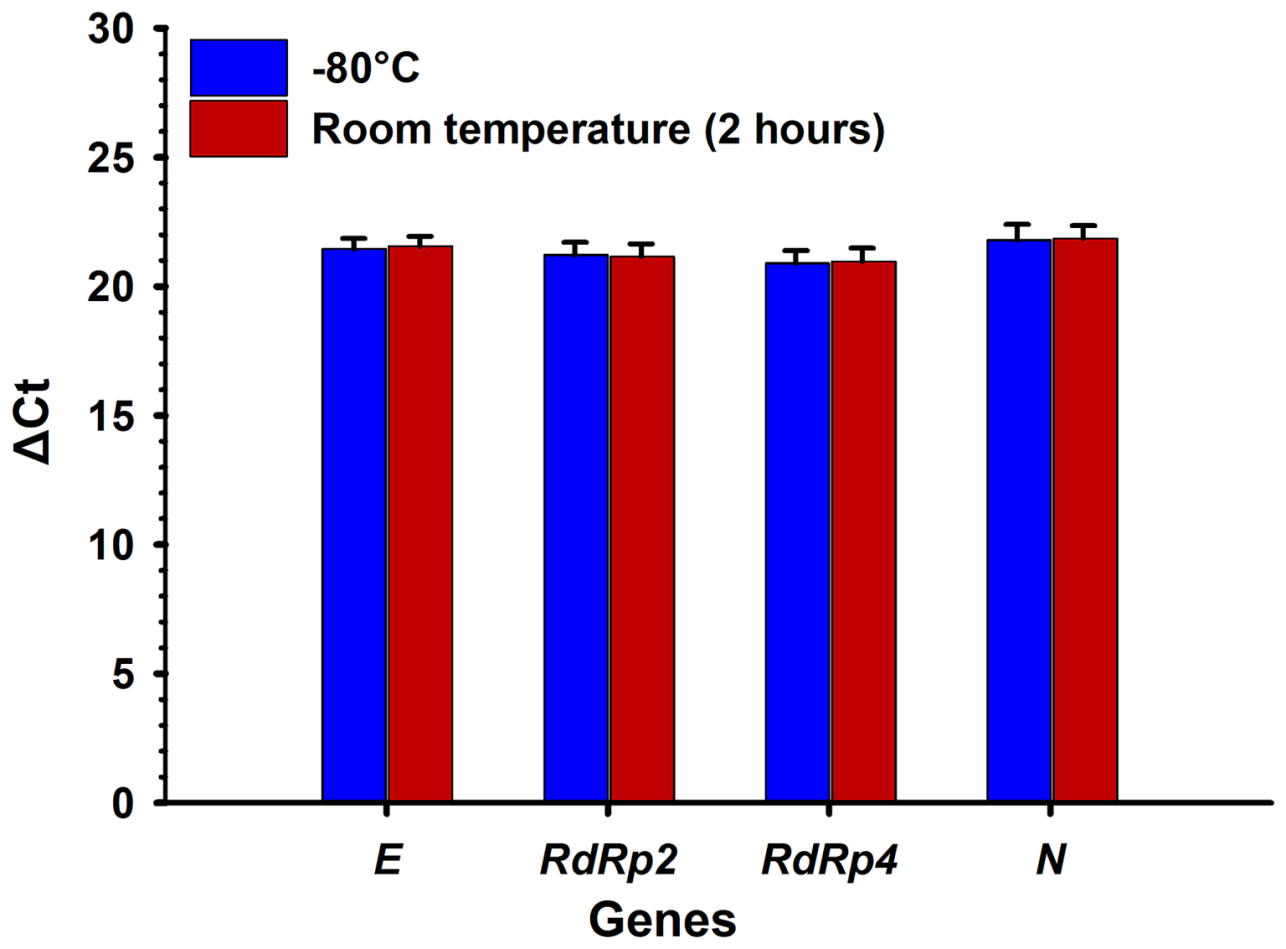
Effect of incubation time at room temperature and −80°C on the detection of SARS-CoV-2 RNA genes. No significant (*p* > 0.05) difference was observed between RNA samples incubated at room temperature for 2 h and at −80°C. ΔCt: delta cycle threshold in the RT-qPCR test. Bars represent the mean of five samples, and error bars indicate the standard deviations. Statistical analysis was performed using a *t*-test.

The results showed that a 2-h room temperature incubation had no significant effect on RNA detectability associated with the *E*, *RdRp2*, *RdRp4*, and *N* SARS-CoV-2 genes compared with the −80°C incubation (*t*-test, two-tailed *p*-values = 0.710, 0.832, 0.837 and 0.871, respectively) (Figure 3).

**Figure 4:**
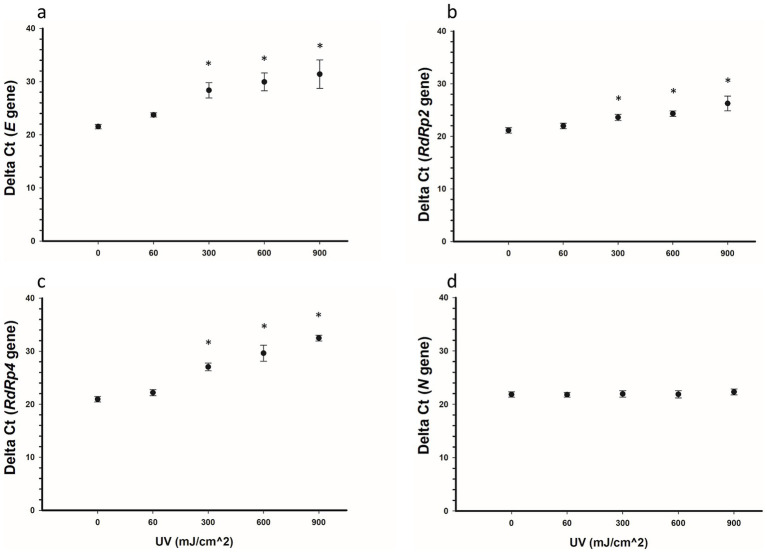
Delta Ct values for SARS-CoV-2 RNA samples irradiated with UV-C irradiation. Five samples were treated with 0, 60, 300, 600, and 900 mJ/cm^2^ UV-C, respectively, and the SARS-CoV-2 RNA *E* **(A)**, *RdRp2* **(B)**, *RdRp4* **(C)**, and *N* **(D)** genes were detected using RT-qPCR. Statistically, the *E*, *RdRp2*, and *RdRp4* genes showed an overall significant increase (*p* < 0.001) in ΔCt values with increased doses of UV-C irradiation, whereas the *N* gene showed no significant (*p* = 0.612) increase. Pairwise comparisons for the *E*, *RdRp2*, and *RdRp4* genes (0 vs. 300, 600, and 900 mJ/cm^2^) were significant (*p* < 0.001) for each gene. Symbols represent the mean ΔCt values of five samples, and error bars indicate the standard deviations. Statistical analysis was conducted using the pairwise multiple comparison test (Bonferroni *t*-test). Star symbols (*) indicate a significant association (*p* < 0.001) compared with the control group (0 mJ/cm^2^).

**Figure 5:**
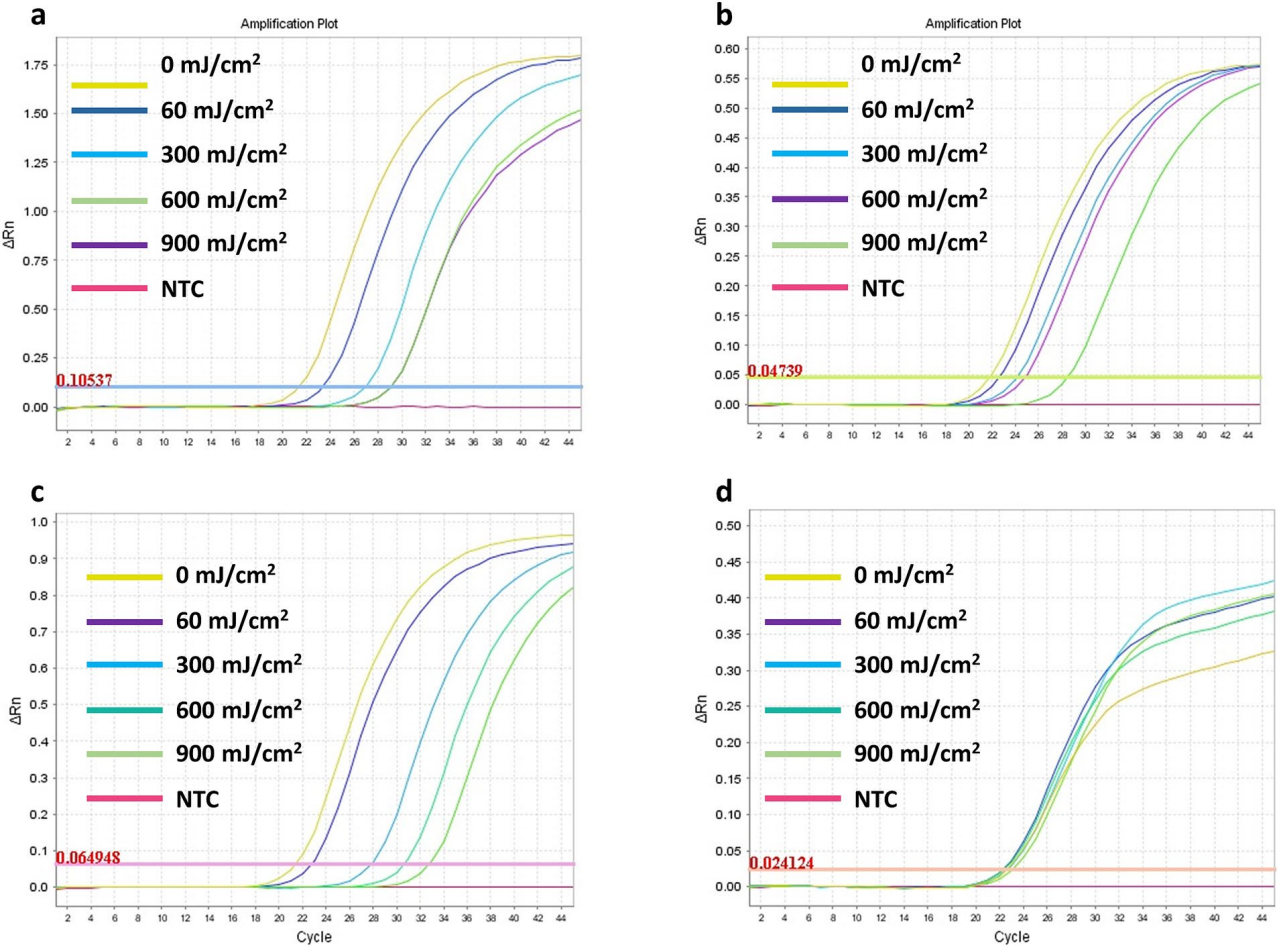
Representative images of the RT-qPCR results for the SARS-CoV-2 RNA *E* **(A)**, *RdRp2* **(B)**, *RdRp4* **(C)**, and *N* **(D)** genes detection following exposure to UV-C irradiation doses ranging from 60 to 900 mJ/cm^2^.

The effect of various UV-C doses (60, 300, 600, and 900 mJ/cm^2^) on the detectability of SARS-CoV-2 RNA was assessed for four viral genes (*E, RdRp2, RdRp4*, and *N*). The results for the respective ΔCt values are presented in Figure 4 and Table 2. Representative images of RT-qPCR results for all SARS-CoV-2 genes are shown in Figure 5. The ΔCt detection threshold increased with increasing UV-C doses, indicating progressive degradation of viral RNA. For the *E*, *RdRp2*, and *RdRp4* genes (Figures 4A–C), the RM-ANOVA indicated an overall significant difference (*p* < 0.001) in mean ΔCt values between the radiation doses. Moreover, a pairwise multiple comparison analysis showed that UV-C at doses of 300, 600, and 900 mJ/cm^2^ resulted in a significant (*p* ≤ 0.001) increase in mean ΔCt values compared with the control (0 mJ/cm^2^) for all three genes (Table 2). However, UV-C irradiation showed no significant (*p* = 0.612) effect on the ΔCt detection threshold of the *N* gene (Figure 4D) or pairwise multiple comparison analysis (Table 2).

**Table 2:** Summary of the effect of UV-C doses on the detectability of SARS-CoV-2 RNA.

Gene	UV-C (mJ/cm^2^)	ΔCt	Overall significant difference) RM-ANOVA (	Pairwise multiple comparisons (Bonferroni *t*-test)
*E*	0	21.5	***p* < 0.001**	
	60	23.7		0.16
	300	28.4		**≤0.001**
	600	29.9		**≤0.001**
	900	31.4		**≤0.001**
*RdRp2*	0	21.1	***p* < 0.001**	
	60	22.0		0.42
	300	23.6		**≤0.001**
	600	24.3		**≤0.001**
	900	26.3		**≤0.001**
*RdRp4*	0	20.9	***p* < 0.001**	
	60	22.2		0.14
	300	27.0		**≤0.001**
	600	29.6		**≤0.001**
	900	32.5		**≤0.001**
*N*	0	21.8	*p* = 0.612	
	60	21.8		1.00
	300	21.9		1.00
	600	21.9		1.00
	900	22.3		0.78

The effect of various UV-C doses (0, 60, 300, 600, and 900 mJ/cm^2^) on the detectability of four SARS-CoV-2 genes (*E*, *RdRp2*, *RdRp4*, and *N*). The ΔCt values represent the delta cycle threshold in the RT-qPCR test. The *p*-values indicate the statistical significance of the difference in ΔCt values compared with the control (0 mJ/cm^2^). Significance was determined by Bonferroni’s *t*-test for pairwise multiple comparisons. Significant *p*-values are in bold font.

## 4 Discussion

The primary aim of this study was to determine the impact of UV-C irradiation on the FE of various FFRs and the stability of SARS-CoV-2 RNA. We evaluated the FE of seven FFR types (3 M-8210, 3 M-1860, Gerson, Medline, Benehal, KN95 “duck shape” and KN95 “molded shape”) following irradiation with various doses of UV-C (0, 60, 120, 180, 240, and 300 mJ/cm^2^) as a potential decontamination method for the reuse of FFRs as a crisis strategy in the event of an N95 mask shortage (42).

The mean initial FE (0 mJ/cm^2^) measurements at all PSs for all FFRs were above 98% (Figure 2). This is in alignment with the standard definition of N95 masks by CDC, which specifies that these masks must filter out at least 95% of airborne particles (5). These results are consistent with our previous study that showed a mean initial FE of ≥98% for Gerson, Medline, KN95 (duck shape), and KN95 (molded shape) FFRs (19). Although the latter study showed that the 3 M-8210 and Benhal FFRs had slightly lower initial FE compared with our results, no significant differences were observed among the FFRs, which is consistent with our results (19). In addition, previous data reported that the overall mean FE (across all PSs) of KN95 masks (FE ≥ 99.74%) was significantly higher (*p* = 0.022) than that of 3 M-8210 masks (FE = 77.1–98.7%) (20); however, the present findings indicate no significant differences among the various FFR types. Consistent with our findings, another study reported a mean initial FE of ≥96.06 for the 3 M-1860, 3 M-8210, and Gerson FFRs (49). The variation in the initial FE results may be attributed to the different procedures used to assess FE, including the NIOSH standard procedure and in-house FE assessments that were widely applied during the COVID-19 pandemic (19). These differences include variations in PSs used in assessments, flow rates, and test filter sampling time (19).

The prospect of decontaminating and recycling FFRs using UV-C was examined even before the global shortage during the SARS-CoV-2 pandemic for several reasons, including cost efficiency, sustainability, emergency preparedness, policy development, and research purposes (50–54). By exploring these aspects, researchers have provided a basis for a wider adoption of UV-C decontamination during pandemics. These studies have provided valuable data to support the emergency use of this technique when FFR supplies become critically low.

Our results showed that the FE for all tested FFRs was >98% following exposure to various doses of UV-C (60, 120, 180, 240, and 300 mJ/cm^2^), with no statistical difference compared with the control (Figure 2). As expected, our results aligned with several studies that showed the resilience of N95 mask filtration capabilities post-UV-C irradiation using different FE assessment procedures and different FFR types (26, 27, 29, 50–53). For example, Fischer and colleagues examined the FE of N95 masks subjected to three cycles of UV-C decontamination with a total dose of 1980 mJ/cm^2^, a dose that was 6.6-fold higher than the dose applied in the present study (27). We found that N95 masks retained comparable FE performance to the control group after two decontamination cycles and maintained acceptable FE performance after three cycles (27). Another study also demonstrated that 20 cycles of UV-C irradiation, with each cycle delivering 1,000 mJ/cm^2^, had no significant effect on the FE of six different FFR models, which remained above 95% (29). Lindsley et al. determined the effect of several UV-C doses (120–950 mJ/cm^2^) on the FE of four types of N95 masks. They found a small decrease in FE (approximately 1.25%) following low to medium UV doses (52); however, the latter study showed that at a very high dose of UV-C (950 mJ/cm^2^), the FE diminished to approximately 90% (52). Another study also demonstrated that UV-C irradiation up to 10,000 mJ/cm^2^ did not affect the integrity of FFRs at PS of 0.4 μm and below (55).

Our results showed that a 2-h incubation at room temperature had no significant impact on the RNA detectability of the *E*, *RdRp2*, *RdRp4*, and *N* SARS-CoV-2 genes compared with incubation at −80°C (Figure 3). These results are consistent with our previous findings that demonstrated the stability of SARS-CoV-2 RNA, particularly the *E* gene, at room temperature and even longer incubation periods of 48 and 96 h (20).

We also assessed the effect of various UV-C doses (60, 300, 600, and 900 mJ/cm^2^) on the detectability of the *E*, *RdRp2*, *RdRp4*, and *N* SARS-CoV-2 RNA genes. Higher UV-C doses resulted in a dose-dependent increase in ΔCt values for the *E*, *RdRp2*, and *RdRp4* genes, indicating significant (*p* < 0.001) degradation of the viral RNA (Figure 4). However, the *N* gene did not show a significant change in ΔCt values (*p* = 0.612), suggesting that UV-C irradiation had no impact on its detectability.

Our results align with those of other studies demonstrating that UV-C can enhance viral inactivation and detectability, although different methodologies were applied (25, 26, 30, 31, 56). Previous studies have primarily used the median tissue culture infectious dose (TCID50) or the *in vitro* SARS-CoV-2 infection assay to evaluate the effect of UV-C on SARS-CoV-2 infectivity. In contrast, our study focused on assessing the viral RNA degradation through RT-qPCR of specific SARS-CoV-2 gene segments. This approach provides insight into how UV-C irradiation affects the viral genome at a molecular level.

Biasin et al. (31) reported that UV-C achieved ≥3-log inactivation and complete inactivation at doses of 3.7 and 16.9 mJ/cm^2^, respectively, using an in vitro SARS-CoV-2 infection assay that measured the copy number replication of SARS-CoV-2 genes. Similarly, another study showed that UV-C doses of 1 and 3 mJ/cm^2^ resulted in an 88.5 and 99.7% reduction in viable SARS-CoV-2 based on the TCID50 assay, respectively, but with no significant difference in copy number of SARS-CoV-2 RNA between pre- and post-UV-C irradiation (30). However, Ozog et al. reported a significantly higher effective dose, defining a UV-C dose of 1,500 mJ/cm^2^ as necessary to decontaminate FFRs from SARS-CoV-2 infection, as measured by the TCID50 assay (26). Of note, there are no specific recommendations on the minimum UV-C dose required to complete SARS-CoV-2 inactivation. CDC reports that a 1,000 mJ/cm^2^ dose can reduce the tested viable viral loads by 99.9% (25).

Notably, our data showed that the *N* gene did not show a significant change in ΔCt values (*p* = 0.612), suggesting that UV-C irradiation had no impact on *N* gene detectability. Interestingly, such results are consistent with that of other studies indicating that the *N* gene of SARS-CoV-2 is more stable and less prone to mutations compared with other genes (57). Abbasi et al. found that the *N* gene has much higher specificity and stability than the *RdRp* gene, making it a superior gene for identifying new cases of SARS-CoV-2 in clinical samples based on RT-qPCR ΔCt values (57). In addition, the *N* gene is known for its conservation and stability, making it a reliable target for diagnostics and vaccine development, as reported by Dutta et al., who demonstrated its immunogenic properties and lower mutation rate compared with other SARS-CoV-2 viral genes (58). This stability is necessary for consistent detection and may explain why the *N* gene shows less degradation following UV-C exposure compared with the *E*, *RdRp2*, and *RdRp4* genes evaluated in the present study.

One of the limitations of this study is that we did not evaluate the fit test, airflow, and electrostatic charge status of FFRs pre- and post-UV-C irradiation and should be considered in future studies. However, Heimbuch et al. (29) performed a fit test on 15 different types of FFRs, including the 3 M-1860 and Gerson masks, for up to 20 cycles of UV-C irradiation (1,000 mJ/cm^2^ per cycle), and found that UV-C irradiation does not affect fit testing and airflow resistance. Another limitation of this study is the use of the AeroTrak particle counter (TSI, Model 9,306) for FE assessment, which, while effective and practical in emergency settings, is less specialized compared to tools such as the TSI 8130 automated filter tester. The one-minute sampling time and its widespread availability in hospitals for environmental validation make it a suitable alternative during crises. Previous work by our team demonstrated its reliability, showing FE exceeding 95% for most FFRs and a percent uncertainty comparable to the manufacturer’s calibration (19). However, the AeroTrak’s capabilities may not fully match the precision of more advanced tools in non-emergency scenarios. Finally, the small sample size of nasopharyngeal swabs, with only five samples used to evaluate the effect of UV-C on SARS-CoV-2 RNA stability, is another limitation of this study. This limitation reflects the logistical challenges and biomaterial shortage during the COVID-19 pandemic. However, the sample size was sufficient to detect statistically significant degradation of the viral RNA genes across multiple UV-C doses. Larger-scale studies are needed to confirm and strengthen these findings.

In conclusion, we demonstrated that UV-C irradiation effectively maintains the FE of various FFRs while significantly degrading the RNA of most tested SARS-CoV-2 genes; however, the *N* gene exhibited remarkable stability under UV-C exposure, consistent with its known conservation characteristics. These findings support the potential use of UV-C as a decontamination strategy for FFRs during PPE shortages.

## Data Availability

The raw data supporting the conclusions of this article will be made available by the authors, without undue reservation.
