# Supplementary material for: Impact of ultraviolet radiation on filtering facepiece respirators and SARS-CoV-2 detection
Source: Front Public Health. 2025 Mar 6;13:1537742. doi: 10.3389/fpubh.2025.1537742 (PMC11922867; doi:10.3389/fpubh.2025.1537742)
Supplement: Supplementary file 1 [file Data_Sheet_1.DOCX]

# Supplementary materials:

| **Target** | **Primer and probes** | **Sequence** |
| --- | --- | --- |
| ***E gene*** | E_Sarbeco_ Forward | ATA TTG CAG CAG TAC GCA CAC A |
|  | E_Sarbeco_Reverse | ACA GGT ACG TTA ATA GTT AAT AGC GT |
|  | E_Sarbeco Probe | ACA CTA GCC ATC CTT ACT GCG CTT CG [5'] TAMRA [3'] BHQ-1 |
| ***RdRp 2*** | RdRp/nCoV_IP2-Forward | ATGAGCTTAGTCCTGTTG |
|  | RdRp/nCoV_IP2-Reverse | CTCC CTTT GTTG TGTT GT |
|  | RdRp/nCoV_IP2-Probe | AGAT GTCT TGTG CTGC CGGT A [5']Hex [3']BHQ-1 |
| ***RdRp 4*** | RdRp/nCoV_IP4-Forward | GGT AAC TGG TAT GAT TTC G |
|  | RdRp/nCoV_IP4-Reverse | CTG GTC AAG GTT AAT ATA GG |
|  | RdRp/nCoV_IP4-Probe | TCA TAC AAA CCA CGC CAG G [5'] FAM [3'] BHQ-1 |
| ***N gene*** | WH-NIC N-Forward | CGTT TGGT GGAC CCTC AGAT |
|  | WH-NIC N-Reverse | CCCC ACTG CGTT CTCC ATT |
|  | WH-NIC N-Probe | FAM-CAACTGGCAGTAACCA-BQH1 |

**Supplementary Table 1.** Genes and their corresponding primers and probes used for detecting the SARS-CoV-2. *E, RdRp2 and RdRp4* genes, which were adapted from the Pasteur protocol, Charité Institute of Virology, Universitätsmedizin Berlin, Germany, https://www.who.int/docs/default-source/coronaviruse/real-time-rt-pcr-assays-for-the-detection-of-sars-cov-2-institut-pasteur-paris.pdf?sfvrsn=3662fcb6_2. *N* gene and its primers were adapted from the Department of Medical Sciences, Ministry of Public Health, Thailand. https://www.who.int/docs/default-source/coronaviruse/conventional-rt-pcr-followed-by-sequencing-for-detection-of-ncov-rirl-nat-inst-health-t.pdf.

**Supplementary Table 2.** RT-qPCR master mix components, volumes, and concentrations.

| **Singleplex master mix** | **Volume (µL)** | **Final concentration** |
| --- | --- | --- |
| RNA sample | 5.0 | Avg 17.7 ng/µl |
| H_2_O PPI | 3.7 |  |
| Reaction mix 2X | 12.5 | 0.3 mM |
| MgSO_4_ (50 mM) | 0.4 | 0.8 mM |
| Forward Primer (10 μM) | 1.0 | 0.4 μM |
| Reverse Primer (10 μM) | 1.0 | 0.4 μM |
| Probe (10 μM) | 0.4 | 0.16 μM |
| SuperScriptIII RT/ Platinum Taq Mix | 1.0 |  |
| Final volume | 25.0 |  |
|  |  |  |

**Supplementary Table 3.** RT-qPCR cycling conditions.

| **Cycle** | **Temperature (°C)** | **Time (minutes)** | **Number of cycles** |
| --- | --- | --- | --- |
| Reverse transcription | 55 | 20 | X1 |
| Denaturation | 95 | 3 | X1 |
| Amplification | 58 | 0.5 | X40 acquisition |
| Cooling | 40 | 0.5 | X1 |
